# Comparison of robot-assisted single-port-plus-one pyeloplasty vs. laparoscopic single-port pyeloplasty in the treatment of ureteropelvic junction obstruction in children

**DOI:** 10.3389/fped.2024.1371514

**Published:** 2024-04-08

**Authors:** Jun Li, Jingyi Chen, Jinfu Jia, Shaohua He, Di Xu

**Affiliations:** ^1^ Shengli Clinical Medical College, Fujian Medical University, Fuzhou, China; ^2^ Department of Pediatric Surgery, Fujian Provincial Hospital, Fuzhou, China

**Keywords:** UPJO, robot, single-port-plus-one, single-port, laparoscopy

## Abstract

**Objective:**

To compare the efficacy of robot-assisted single-port-plus-one pyeloplasty (RSPY) and laparoscopic single-port pyeloplasty (LSPY) in the treatment of children with ureteropelvic junction obstruction (UPJO).

**Methods:**

The clinical data of 47 children who underwent surgery for UPJO at the Department of the Pediatric Surgery of the Fujian Provincial Hospital from October 2020 to September 2022 were analyzed retrospectively. Of these 47 children, 27 received RSPY while 20 underwent LSPY. The baseline data, operation time, intraoperative anastomosis time, intraoperative blood loss, postoperative hospital stay, complications, total cost, preoperative and postoperative renal parenchymal thickness (PT), anteroposterior renal pelvis diameter (APD), and differential renal function (DRF) of the two groups were compared to evaluate the clinical efficacy of the two surgical methods.

**Results:**

The results showed that both surgical techniques were successful and no patient transitioned to open surgery. There was no significant difference between the two groups in baseline data, intraoperative blood loss, complications, APD, and PT 6 months after surgery. There was also no significant difference in APD, PT, and DRF 12 months after surgery (all *P* > 0.05). Compared with the LSPY group, the RSPY group had shorter operation time [(153.04 ± 14.44) vs. (189.90 ± 32.59) min, *t* = −5.24, *P* < 0.05], less intraoperative anastomosis time [(68.81 ± 16.80) vs. (97.45 ± 11.99) min, *t* = −6.49, *P* < 0.05], shorter postoperative hospital stay [(5.96 ± 1.34) vs. (9.00 ± 1.33) d, *t* = −7.68, *P* < 0.05], but higher total cost [(57,390 ± 7,664) vs. (30,183 ± 4,219) yuan, *t* = 14.32, *P* < 0.05].

**Conclusion:**

Compared with LSPY, RSPY achieves equivalent efficacy in the treatment of UPJO in children and has certain advantages by shortening the operation time, intraoperative anastomosis time, and postoperative hospital stay. However, its cost burden is heavy, and appropriate cases need to be selected for popularization and application.

## Introduction

Ureteropelvic junction obstruction (UPJO) is a common cause of congenital hydronephrosis in children and is characterized by intrinsic, extrinsic, or secondary obstruction (1), with severe cases requiring surgical intervention. Open pyeloplasty (OP) was once considered the gold standard for the treatment of pediatric UPJO and had a success rate of more than 90% (2). Following the development of minimally invasive surgical techniques the first laparoscopic pyeloplasty (LP) was reported by Schuessler in 1993 (3). Since then, LP has become the preferred procedure (4). The first robot-assisted laparoscopic pyeloplasty (RALP) was performed in 1999 by Yanke (5). Based on LP and RALP, single-incision laparoscopic surgery (SILS) and single-incision-plus-one laparoscopic surgery (SILS + 1) were developed for robot-assisted single-port-plus-one pyeloplasty (RSPY) and laparoscopic single-port pyeloplasty (LSPY), respectively. Only a small number of studies have compared RSPY and LSPY and therefore we undertook this retrospective study to analyze the clinical data of 47 children with UPJO and compared the efficacy of the two techniques.

## Materials and methods

### Patients and design

This study was approved by the Institutional Review Board of Fujian Provincial Hospital.The study selected 47 children with UPJO who had undergone surgical treatment at the Department of Pediatric Surgery of the Fujian Provincial Hospital between October 2020 and September 2022. The natural grouping was based on the surgical method selected by the parents of the children. Of the 47 children, 27 had received RSPY and 20 underwent LSPY. The Robotic platform used was the DaVinci Xi.The family members of all the patients were informed of the details of the study and signed the consent form. The same surgeon performed the procedure. All the patients underwent urological ultrasound, magnetic resonance urography (MRU), renal static imaging, a diuretic renogram, and voiding cystourethrography (VCUG) before surgery. Gender, age, affected side, weight, follow-up time, renal parenchymal thickness (PT), anteroposterior renal pelvis diameter (APD), and differential renal function (DRF) were recorded. As shown in Table 1, the baseline data of the two groups were comparable (*P *> 0.05).

**Table 1:** Preoperative baseline data in the two groups.

	RSPY group	LSPY group	*t*/*χ*^2^	*P*
Gender (*n*)			0.01	0.959
Male	16	12		
Female	11	8		
Age (year)	6.61 ± 3.51	6.55 ± 3.50	0.07	0.948
Affected side (*n*)			0.21	0.886
Left	17	13		
Right	10	7		
Body weight (kg)	26.55 ± 13.0	25.17 ± 10.26	0.39	0.697
Preoperative APD (mm)	36.00 ± 8.59	34.79 ± 7.91	0.50	0.623
Preoperative DRF (%)	35.03 ± 7.39	31.79 ± 6.08	1.60	0.117
Preoperative PT (mm)	7.30 ± 2.63	7.53 ± 2.40	−0.31	0.759
Follow-up time (months)	12.78 ± 4.68	11.35 ± 4.15	1.09	0.284

Inclusion criteria: Initial surgery; recurrent urinary tract infection, abdominal pain, abdominal mass; renal function below 40% on renal static imaging; mechanical obstruction on a diuretic renogram; a decrease in renal function >10% during the follow-up.

Exclusion criteria: Secondary surgery; uncontrolled acute urinary tract infection; combined with either duplication of kidney and other urinary tract anatomical malformations, or with vesicoureteral reflux, ureterovesical junction obstruction or other causes of hydronephrosis.

### Surgical techniques

#### RSPY

**Figure 1:**
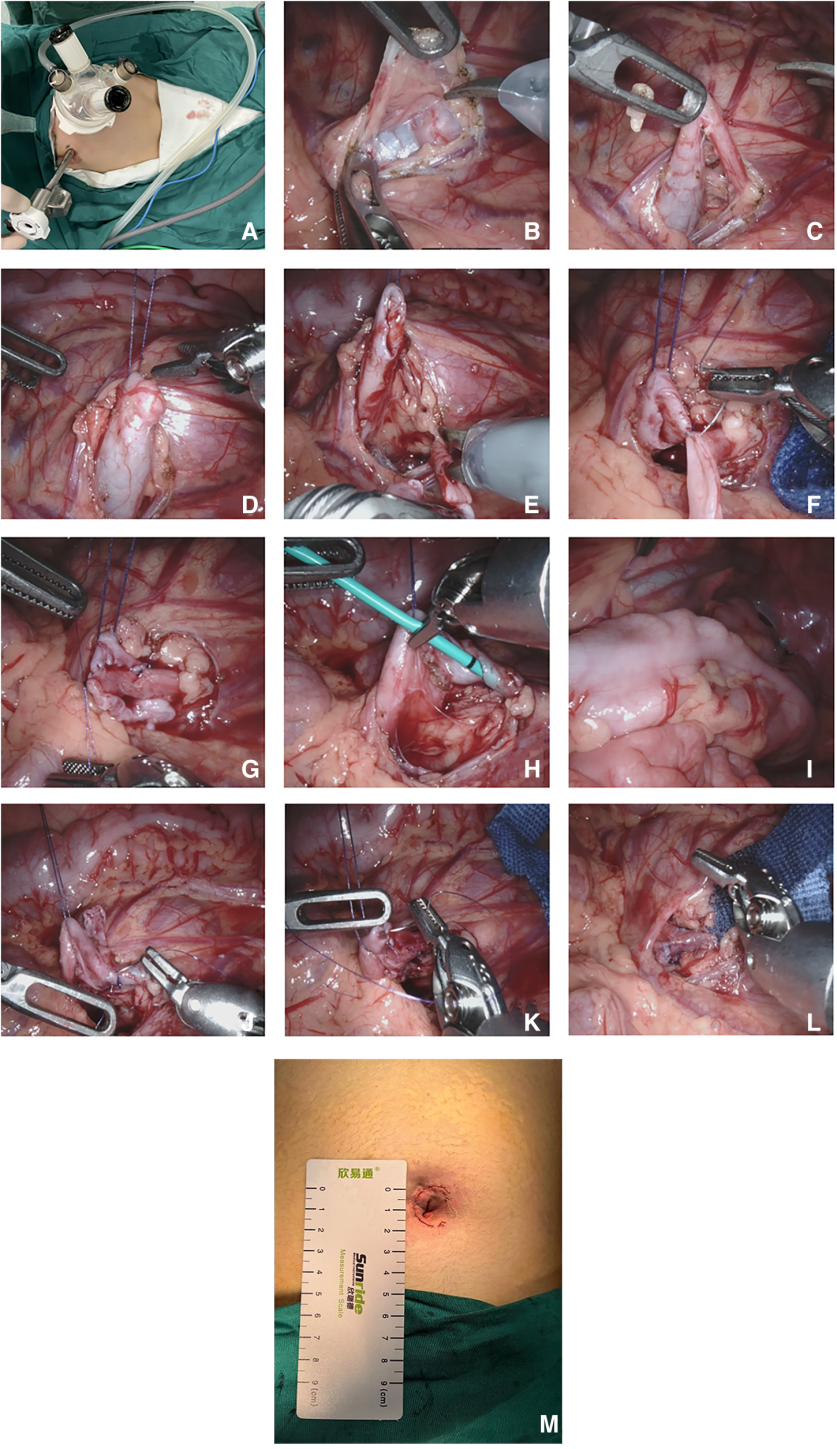
Robot-assisted single-port-plus-one laparoscopic pyeloplasty. (**A**) The location of the trocar; (**B**) Transcolonic medial mesenteric approach; (**C**) Exposure of the narrow UPJ; (**D**) Suspension of the renal pelvis using 4-0 Vicryl sutures; (**E**) Resection of the narrow UPJ and longitudinal incision of the distal ureter; (**F**) Suturing of the lowest point of the renal pelvis and the ureter; (**G**) Suturing of the posterior wall of the anastomosis; (**H**) Insertion of a ureteral stent tube through the anastomosis; (**I**) Confirmation that the ureteral stent tube was in the bladder; (**J**) Suturing of the anterior wall of the anastomosis; (**K**) Suturing of the renal pelvis; (**L**) Newly formed ureteropelvic junction; (**M**) The postoperative single port incision.

The child was placed in the supine position, with the affected side slightly elevated and the operating bed tilted by 30 to 45 degrees towards the healthy side. The bilateral upper limbs were placed in a “pitching” position and the bilateral lower limbs slightly opened. All stress areas were fixed with sponge pads and bandages used to fix the limbs. An arc-shaped incision was made at the umbilical edge with the pedunculated side facing the affected kidney that allowed entry into the abdominal cavity layer by layer. A disposable, multi-channel, single-port laparoscopic trocar was then positioned for insertion of an 8.0-mm endoscopic sheath (connected to the 3rd robotic arm), main operating sheath (connected to the 2nd robotic arm), and auxiliary operating instruments. An 8-mm incision was made in the flank 6 cm from the umbilicus to place an operating sheath connected to the 4th robotic arm (Figure 1A). A pneumoperitoneum tube was connected to establish the pneumoperitoneum and maintain its pressure of 8–12 mmHg.

Using a mesenteric approach (Figure 1B), the affected renal pelvis and upper ureter were mobilized and exposed to identify the site of obstruction (Figure 1C). The upper pole of the renal pelvis was suspended and pulled by a 4-0 Vicryl suture through the abdominal wall (Figure 1D). The narrow ureteropelvic junction was resected, and about 2.0 cm of the lateral ureteral wall incised longitudinally (Figure 1E). The lowest point of renal pelvis and distal ureter were determined and sutured point to point with 6-0 PDS PLUS (Figure 1F). The posterior wall of the anastomosis was then sutured continuously (Figure 1G). A ureteral stent was placed antegrade through the anastomosis (Figure 1H) to verify that it had reached the bladder (Figure 1I). The anterior wall of the anastomosis (Figure 1J) and the excess opening of the renal pelvis valve (Figure 1K) were sutured continuously to form a new anastomosis (Figure 1L). After the anastomosis was finished, furosemide was administered to make sure there was no urine leakage at the anastomosis site. After washing the wound with warm physiological saline, the intra-abdominal effusion was aspirated to confirm no active bleeding in the surgical field. Absorbable sutures were used to intermittently suture the mesenteric fissure without an indwelling abdominal drainage tube. The docking was released, the robotic platform was removed, and the incision (Figure 1M) was sutured layer by layer (6).

#### LSPY

The surgical steps were basically the same as for the RSPY, except for the absence of the additional lateral abdominal operation sheaths, docking with robot surgical platforms, and installation and disassembly of the robotic arms.

### Data collection

The patients' perioperative characteristics including operation time, intraoperative anastomosis time, intraoperative blood loss, postoperative hospital stay, complications, and total costs were collected. Aiso the postoperative PT, APD and DRF data were recorded.

### Statistical analysis

All data were analyzed using SPSS 22.0 statistical software. The measurement data with a normal distribution were expressed as $\bar{x}\pm s$ and a *t*-test used for comparison between the two groups. The rank-sum test was used for comparison of measurement data that did not conform to a normal distribution. The counting data were expressed as numbers, and compared using the chi-square test. A *P*-value < 0.05 indicated a statistically significant difference.

## Results

### Perioperative data

Both groups of cases underwent successful surgery without transitioning to open surgery. In the RSPY group, the surgical time was 153.04 ± 14.44 min, the intraoperative anastomosis time was 68.81 ± 16.80 min, the intraoperative bleeding volume was 3.29 ± 0.84 ml, and the postoperative hospitalization time was 5.96 ± 1.34 days. One case developed a postoperative urinary tract infection. The total cost of the operations was 57,390 ± 7,664 yuan.

In the LSPY group, the surgical time was 189.90 ± 32.59 min, the intraoperative anastomosis time was 97.45 ± 11.99 min, the intraoperative blood loss was 6.78 ± 1.81 ml, and the postoperative hospital stay was 9.00 ± 1.33 days. Three patients developed postoperative urinary tract infections. The total cost of the operations was 30,183 ± 4,219 yuan. The operation time, intraoperative anastomosis time, and postoperative hospital stay were significantly lower in the RSPY group than in the LSPY group (*P* < 0.05). However, the total cost of the operations in the RSPY group was substantially higher than that of the LSPY group (*P* < 0.05). As shown in Table 2, intraoperative blood loss and postoperative complications were roughly the same in both groups (*P* > 0.05).

**Table 2:** Perioperative data between the two groups.

	RSPY group	LSPY group	*t*/*χ*^2^	*P*
Surgical time (min)	153.04 ± 14.44	189.90 ± 32.59	−5.24	<0.001
Intraoperative anastomosis time (min)	68.81 ± 16.80	97.45 ± 11.99	−6.49	<0.001
Intraoperative blood loss (ml)	3.29 ± 0.84	6.78 ± 1.81	−8.84	0.120
Postoperative hospital stay (d)	5.96 ± 1.34	9.00 ± 1.33	−7.68	<0.001
Complications (*n*)	1	3	0.71	0.399
Total cost (yuan)	57,390 ± 7,664	30,183 ± 4,219	14.32	<0.001

### Postoperative follow-up indicators

As shown in Table 3, there was no statistically significant difference in APD or PT at 6 and 12 months postoperative, and DRF at 12 months postoperative between the two groups (*P* > 0.05).

**Table 3:** Postoperative follow-up indicators between the two groups.

	RSPY group	LSPY group	*t*	*P*
6 months postoperative				
APD (mm)	25.66 ± 7.07	24.32 ± 7.33	0.63	0.529
PT (mm)	9.65 ± 2.47	10.18 ± 2.59	−0.71	0.482
12 months postoperative				
APD (mm)	23.51 ± 7.54	22.42 ± 8.14	0.47	0.638
PT (mm)	11.17 ± 2.66	12.02 ± 2.56	−1.09	0.281
DRF (%)	42.90 ± 5.07	40.08 ± 4.96	1.42	0.164

## Discussion

Anderson-Hynes first reported OP. With the development of laparoscopic techniques, LP has steadily emerged as the new gold standard. Compared with OP, LP causes smaller scars and milder pain and results in a more rapid recovery and shorter hospital stays (7). RALP was carried out for the first time in 1999 following the approval of the da Vinci robotic surgical platform for clinical use (5). Gettman (8) reported nine cases who underwent RALP in 2002, with a success rate of 100%. However, LP and RALP required 3–4 abdominal incisions and progress still needed to be made for improving aesthetics. A small number of abdominal scars or even no scars are currently hotspots in minimally invasive pediatric surgery, a requirement that has led to the development of RSPY and LSPY.

LSPY is performed by placing a single-port multi-channel device in the umbilical region. Compared with multi-port laparoscopy, LSPY is accomplished through a single umbilical incision that can be concealed, thereby conforming with the concept of minimally invasive surgery. However, the single-port laparoscopy is technically difficult due to the device losing the principle of triangular distribution, making it difficult to form an effective operational triangle. Moreover, there is a “coaxial effect”, with the device prone to collision and interference during the operation. Furthermore, the narrow space is not conducive to delicate operations like traction, separation, and especially suturing (9). White (10) reported eight cases who underwent LSPY, none of who converted to open surgery, with an average surgical time of 233 min. However, one case developed an incisional hernia after surgery. Tracy (11) compared the efficacy of LSPY and LP for treating UPJO and showed no significant difference in postoperative complications and hospital stay between the two groups, leading them to concluded that LSPY and LP had equivalent effectiveness for treating UPJO. Fu (12) reported 15 cases of LSPY, with an average surgical time of 90 min and a follow-up cure rate of 100%, while Yang (9) reviewed eight surgical cases, with an average surgical time of 153 min and no complications in which the patient's symptoms were largely improved compared with their preoperative condition. Zheng (13) performed LSPY on 135 infants aged less than 3 months with severe hydronephrosis and reported an average surgical time of 81 min and average bleeding volume of 7.8 ml, with 127 cases showing significant improvement in hydronephrosis and 9 cases having no significant improvement. These studies reported differences in the operation time for LSPY, possibly due to the learning curve and different surgical techniques and habits of the surgeons.

Compared with traditional laparoscopic surgery, robotic surgery has significant advantages. First, it provides a three-dimensional stereoscopic field of view and a 10-fold magnification of the conventional field, which is more conducive to accurate positioning. Second, robotic surgical instruments have more minor pivot effects and higher degrees of freedom, with the degree of bending and rotation of the instruments far exceeding the limit of human hand joints. Third, the vibration filtering feature of the robotic system makes the operation more stable. Fourth, robotic surgery has better ergonomics that helps alleviate the fatigue of surgeons during surgery. Fifth, performing the refined operation in a limited space reduces secondary injuries (6, 14–17). Finally, the learning curve of robot surgery is short, and is significantly shorter in surgeons with considerable LP experience. Therefore, robotic surgery is more suitable for complex body-cavity procedures, especially reconstructive surgery. The abdominal cavity space of children is small, which requires more refined procedures, and the robotic arm can be operated flexibly in deep, narrow areas (18–22).

Following the introduction of da Vinci robotic Xi surgery in our department in October 2020, RSPY has been used successively in the radical resection of choledochal cysts and pyeloplasty. This surgery separated the main operating port and placed the 2nd robotic arm (connected to a unipolar shear), 3rd robotic arm (connected to an 8 mm eyepiece), and auxiliary endoscopic instruments in a single-port multichannel device. An additional port was added 6 cm from the umbilicus on the abdomen side to place a Cardier connected to the 4th robotic arm. Therefore, the distance between the 2nd and 3rd robotic arms was 4–5 cm, and between the 3rd and 4th robotic arms was 3–4 cm (23, 24). In addition, these devices when combined with endoscopic suspension of the renal pelvis, fully exposed the surgical field of view, increased the operating space, and significantly reduced the mutual collision and interference between instruments. The additional port also allowed the 2nd and 4th robotic arms to be manipulated by the surgeon and the auxiliary port by the assistant, thereby forming a triangular structure that facilitated microscopic mobilization and anastomosis (25).

The current study showed that the operation time was shorter in the RSPY group than in the LSPY group, with this difference being statistically significant (*P* < 0.05). Consistent with our findings, Chen (22) reported that the operation time of RSPY and LSPY in the treatment of pediatric UPJO was 169.80 ± 18.88 min and 212.0 ± 98.27 min, respectively, with the operation time of the RSPY group being significantly shorter than that of the LSPY group (*P* < 0.05). They also showed that the time for placing ureteral stent tubes in the RSPY and LSPY groups was 2.90 ± 0.99 min and 3.40 ± 1.14 min, respectively, and although this difference was not statistically significant, it was suggested that robotic surgery was also superior for ureteral stent placement. The intraoperative anastomosis time was also significantly shorter in the RSPY group than in the LSPY group (*P* < 0.05), which reflected the benefits of a robotic surgical platform for fine sutures. The majority of published studies on RALP have mentioned the great advantage of robotic arms for suturing (7), with the robot platform having stable operation and flexible robotic arms that can be operated in multiple dimensions. In addition, the robotic platform makes it easier to perform deep abdominal knotting than that achieved using single-port laparoscopy, by reducing instrument collisions and reducing the anastomosis time. The current study confirmed these advantages. With the continuous accumulation of surgical experience, we have become more skilled in docking robotic platforms, installing robotic arms, and placing surgical instruments, although it is still possible to further shorten the surgical time. Our study also showed that the postoperative hospital stay was statistically shorter in the RSPY group than in the LSPY group. We speculate that the reason may be the robotic surgery was more precise, with less damage to the surrounding tissues, minimal secondary damage, less inflammatory reaction and exudation in the surgical area, and faster postoperative repair of abdominal tissue (26). What's more, the robotic surgery has a short operation time, minimal disturbance to the intestines, and the fulcrum effect of the robotic arm is small, causing minimal damage to the human body. These lead to the differences of postoperative hospital stay. In addition, both groups of cases achieved satisfactory treatment effects and outcomes with no significant difference in postoperative complications between the two groups (*P* < 0.05). All urinary tract infections were cured after anti-infection treatment, the UTI occur between discharge and the removal of ureteral stents next time admitted. The reason for the infection is due to the stent being a foreign object, which some children cannot tolerate. After extubation, no infections occurred in the children. And there was no significant difference observed in APD, PT, and DRF between the two groups at 6 months and 12 months after the operation (*P* < 0.05). Taken together, these results indicated that the short-term efficacy of the two surgical methods was equivalent and that RSPY achieved comparable efficacy as LSPY and helped patients achieve better perioperative benefits.

Compared with LSPY, RSPY has inherent shortcomings. First, RSPY has a higher cost, which places a heavy burden on the families of patients. The current study showed the RSPY group had higher costs than the LSPY group, with this difference being statistically significant (*P* < 0.05). However, this situation is expected to improve following introduction of the domestic robotic surgery platform. Second, the robotic surgical system lacks a mechanical feedback system and therefore the surgeon has no tactile sensation when stitching and knotting and only relies on visual feedback to compensate. Third, the body position cannot be changed arbitrarily once the robot surgical platform is successfully docked. Finally, robotic surgery has a larger chance of mechanical failure compared with traditional laparoscope surgery, which might result in an inability to continue the surgery.

The current study had shortcomings as it was a single-center retrospective study, with the sample size of the two groups being insufficient to provide a high level of statistical power. The grouping was also formed by the parental selection of the surgical methods, which introduced some selection bias. Large-sample, multicenter, and prospective studies are therefore required to clarify the long-term efficacy of the two surgical techniques.

In conclusion, compared with LSPY, RSPY achieves equivalent efficacy for treating pediatric UPJO and has certain advantages by shortening surgical time, intraoperative anastomosis time, and postoperative hospitalization days. However, its cost burden is heavy, and suitable cases need to be selected for the promotion and application of the surgical technique.

## Data Availability

The raw data supporting the conclusions of this article will be made available by the authors, without undue reservation.
